# Development of an interpretable machine learning model for early prediction of aortic stiffness risk in health examination populations

**DOI:** 10.3389/fcvm.2025.1730409

**Published:** 2026-01-07

**Authors:** Zhensen Zhou, Xiao Sun, Hongmei Qian, Zuchang Ma, Fuying Kong, Ting Li, Wanqiu Zhang, Yuxia Li

**Affiliations:** 1 Department of Gastroenterology, The First Affiliated Hospital of Anhui Medical University, Hefei, Anhui, China; 2 Institute of Intelligent Machines, Hefei Institutes of Physical Science, Chinese Academy of Sciences, Hefei, China; 3 Health Management Center, The Hefei Cancer Hospital, Chinese Academy of Sciences, Hefei, China

**Keywords:** aortic stiffness, carotid-femoral pulse wave velocity, early diagnosis, machine learning, SHAP (SHapley additive exPlanation)

## Abstract

**Background:**

Carotid-femoral pulse wave velocity (cfPWV) is the gold standard for assessing aortic stiffness, but its complexity, time consumption, and privacy concerns limit its application in routine health examinations. This study aimed to develop an interpretable machine learning model based on readily accessible indicators, providing an alternative screening tool for institutions unable to perform cfPWV measurements.

**Methods:**

A total of 261 participants were enrolled as the development cohort and randomly divided into training and testing sets (7:3 ratio), with 101 additional participants as an independent external validation set. Aortic stiffness was defined as cfPWV ≥10 m/s. Features were selected by combining univariate logistic regression, recursive feature elimination with random forest (RFE-RF), and least absolute shrinkage and selection operator (LASSO) methods. Six machine learning models were constructed: logistic regression (LR), random forest (RF), extreme gradient boosting (XGBoost), support vector machine (SVM), Naive Bayes (NB), and K-nearest neighbours (KNN). Internal validation was performed using 5-fold cross-validation, and performance was evaluated using area under the receiver operating characteristic curve (AUC), calibration curves, and decision curve analysis (DCA). The optimal model was validated on the external set, and interpretability was analyzed using SHapley Additive exPlanations (SHAP). Subsequently, the model was deployed as an interactive, web-based application utilizing the Streamlit framework in Python.

**Results:**

Seven key variables were selected: Age, body mass index (BMI), mean arterial pressure (MAP), fasting blood glucose (FBG), high-density lipoprotein cholesterol (HDL-C), glomerular filtration rate (GFR), and aspartate aminotransferase (AST). The XGBoost model achieved the best performance with an AUC of 0.903 (95% CI: 0.830–0.975) on the testing set and a mean AUC of 0.979 (95% CI: 0.960–0.997) in 5-fold cross-validation on the training set. External validation demonstrated robust generalizability (AUC = 1.000). DCA indicated a favourable clinical net benefit, and SHAP analysis quantified feature contributions.

**Conclusion:**

This study developed a high-performance XGBoost model based on routine health examination indicators and further implemented an accessible web-based calculator to support clinical decision-making in settings without direct access to cfPWV measurement. The calculator is available at: https://lgezijo5wyivqrnt2zrkcm.streamlit.app/.

## Background

Cardiovascular disease (CVD) represents a significant and growing global public health burden. The American Heart Association's 2024 report projects that CVD prevalence in the United States will rise from 11.3% in 2020 to 15.0% by 2050, affecting over 45 million adults (1). Aortic stiffness has emerged as a critical predictor of cardiovascular event risk (2) and an independent determinant of cardiovascular events and all-cause mortality (3). Carotid-femoral pulse wave velocity (cfPWV), the gold standard for assessing aortic stiffness, quantifies arterial compliance by measuring the speed at which pressure waves travel through the aorta (4). Due to its substantial predictive value and reproducibility, cfPWV has been incorporated into multiple international clinical guidelines for assessing arterial function and early cardiovascular risk stratification (5–7).

According to the joint guidelines from the European Society of Cardiology (ESC) and the European Society of Hypertension (ESH), a cfPWV ≥10 m/s serves as the diagnostic threshold for increased aortic stiffness and is considered a marker of subclinical organ damage (8). Given that arterial dysfunction precedes structural changes in the pathophysiological progression of vascular disease (9), cardiovascular prevention strategies must shift from passive management of late-stage structural damage to proactive intervention targeting early functional abnormalities. Therefore, early identification and intervention of aortic stiffness may delay the progression of arterial structural damage and significantly reduce the risk of adverse cardiovascular events.

However, although the clinical value of cfPWV has been widely recognized, its application in routine health management is still limited due to the high cost of measurement equipment, the relatively complex measurement process and privacy issues. In addition, previous cross-sectional studies on cfPWV mostly originated from hospital cases, which involved selection bias, making it difficult to generalize to early asymptomatic populations. In terms of prediction model construction, traditional statistical methods have both advantages and disadvantages compared to complex machine learning algorithms. Conventional methods (e.g., logistic regression) have good interpretability, but are often considered to have limited predictive performance. Ensemble learning methods (e.g., random forest, gradient boosting tree), although theoretically able to capture complex nonlinear relationships, face challenges such as overfitting and poor interpretability. Here, we compared multiple machine-learning algorithms to predict aortic stiffness risk using routinely collected health examination data and identified the optimal-performing model. To enhance transparency and clinical interpretability, we applied SHapley Additive exPlanations (SHAP) to quantify feature contributions at both global and individual levels. Based on the selected model, we further developed a web-based risk calculator, providing an accurate and interpretable screening tool for settings where cfPWV measurement is unavailable.

## Methods

### Study subjects

**Figure 1:**
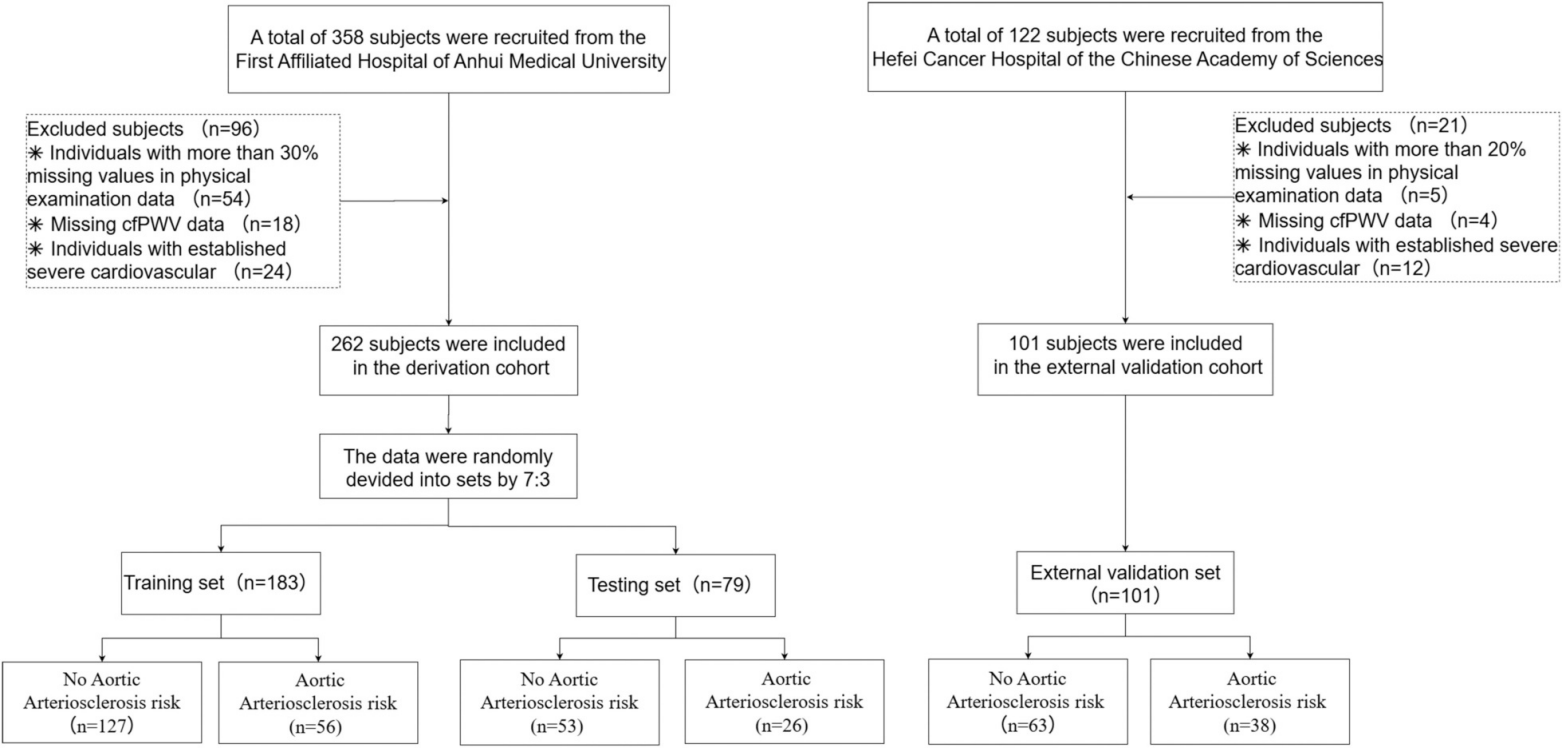
The participant selection process.

This cross-sectional study enrolled individuals who underwent routine health examinations at the Health Management Centre of the First Affiliated Hospital of Anhui Medical University between June and December 2023. Inclusion criteria were: (1) age ≥18 years; and (2) good compliance and provision of written informed consent. Exclusion criteria included: (1) history of psychiatric disorders; (2) diagnosed severe cardiovascular or cerebrovascular disease, hepatic or renal dysfunction, or other major organic diseases; (3) severe peripheral arterial occlusive disease affecting cfPWV measurement (e.g., severe stenosis or occlusion of the carotid or femoral arteries); (4) other conditions deemed unsuitable for enrollment by investigators (e.g., pregnancy, inability to palpate arterial pulsation at measurement sites); and (5) missing data exceeding 20% of health examination parameters. The participant selection process is shown in Figure 1. A total of 261 participants constituted the model development cohort for training (70%) and testing (30%). To assess model generalizability, an external validation cohort of 101 participants was independently recruited from the Health Management Centre, Hefei Cancer Hospital, and the Chinese Academy of Sciences (February–May 2024).

### Data collection

Based on ESC/ESH guidelines, cfPWV ≥10 m/s was used to define increased aortic stiffness, creating a binary outcome variable (8). Baseline characteristics were collected, including Age, sex, body mass index (BMI), hypertension, diabetes, systolic blood pressure (SBP), diastolic blood pressure (DBP), and smoking status [defined as smoking ≥1 cigarette per day within 30 days before the survey (10)]. Blood pressure was measured three times at the right brachial artery in the supine position using an OMRON M6 device (≥1-minute intervals). Mean arterial pressure (MAP) was calculated from the average of three consecutive measurements: MAP = (SBP + 2 × DBP)/3. After an overnight fast (≥8 h), venous blood samples were collected from the antecubital vein. Laboratory parameters included total cholesterol (TC), low-density lipoprotein cholesterol (LDL-C), high-density lipoprotein cholesterol (HDL-C), triglycerides (TG), fasting blood glucose (FBG), glomerular filtration rate (GFR), eosinophils, basophils, urea, uric acid (UA), haemoglobin (Hb), alanine aminotransferase (ALT), aspartate aminotransferase (AST), platelet count (PLT), and creatinine.

### CfPWV measurement

Carotid-femoral pulse wave velocity was assessed using a SphygmoCor XCEL device (AtCor Medical, Sydney, Australia). After 10 min of supine rest, cfPWV was measured at the sites of maximal pulsation of the right carotid and femoral arteries (11). Participants remained silent and awake during measurement. The direct distance between measurement sites was recorded using a tape measure. The corrected carotid-femoral distance was calculated as measured distance × 0.8 (11). cfPWV was automatically computed from 10 stable waveforms using the formula: cfPWV (*m*/*s*) = (*L* × 0.8)/Δ*t*, where *L* is the direct distance (*m*) and Δ*t* is pulse transit time (*s*) (12). The mean of two measurements was used; if the difference exceeded 0.5 m/s, a third measurement was obtained and the median value was recorded.

### Data preprocessing

Missingness was assessed for all variables. Variables with more than 20% missing data were excluded. Only predictor variables were imputed; the outcome variable was not imputed. Missing continuous values were imputed using predictive mean matching (PMM), which preserves the original data distribution and avoids the variance shrinkage associated with mean imputation. Missing categorical values were imputed using classification and regression trees (CART), which flexibly captures nonlinear and interaction effects without requiring parametric assumptions. Together, PMM and CART provide more distribution-preserving and robust imputations than simple mean/mode imputation or parametric multiple imputation. Outliers were identified using the interquartile range (IQR) method (values <Q1 − 1.5 × IQR or >Q3 + 1.5 × IQR) (13). Implausible values were set to missing prior to imputation, while plausible extreme values were retained.

### Feature selection

**Figure 2:**
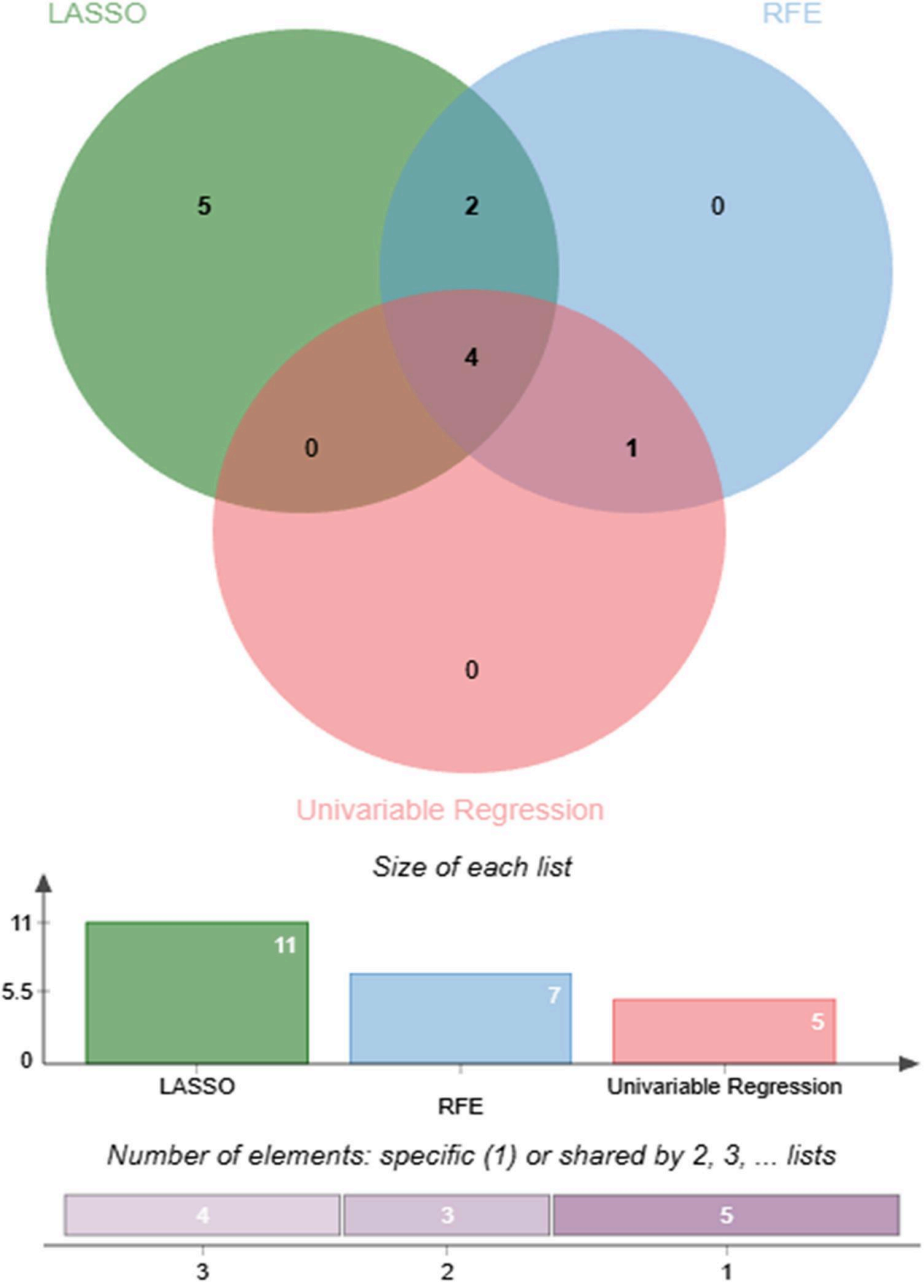
Comparison and consensus of features selected by three different methods.

The preprocessed data were randomly divided into training (70%) and testing (30%) sets. To reduce potential bias arising from manual feature selection and to enhance the robustness of the selection process, three complementary feature selection methods were employed: univariate logistic regression, Least Absolute Shrinkage and Selection Operator (LASSO) regression, and Recursive Feature Elimination (RFE). These approaches evaluate candidate variables from the perspectives of statistical association, regularization-based shrinkage, and model performance optimization, respectively. A consistency-based strategy was adopted, whereby only variables selected by at least two of the three methods were retained in the final feature set, as shown in Figure 2. This approach leverages the strengths of multiple selection techniques and improves the reliability and stability of the selected features. To further ensure the suitability of the selected variables, multicollinearity was assessed using the Variance Inflation Factor (VIF).

### Model construction and validation

Six machine learning algorithms were developed: logistic regression (LR), random forest (RF), extreme gradient boosting (XGBoost), support vector machine (SVM), Naïve Bayes (NB), and k-nearest neighbours (KNN). Hyperparameters were optimized using grid search with 10-fold cross-validation. The algorithm with the highest area under the receiver operating characteristic curve (AUC) on the testing set was selected as the final model. To assess model robustness and generalizability, 5-fold cross-validation was performed on the training set, and external validation was conducted on an independent cohort (*n* = 101). Model interpretability was evaluated using SHAP to quantify feature contributions and their directional effects on predictions. Model performance was assessed using the following metrics: AUC, accuracy, recall, specificity, *F*1 score, positive predictive value (PPV), and negative predictive value (NPV). Calibration performance was evaluated using calibration curves to assess the agreement between predicted and observed risks, supplemented by model-agnostic calibration error metrics for non-probabilistic models. In addition, the clinical utility of each model was assessed using decision curve analysis (DCA), which compares the net benefit of model-guided decisions across a range of clinically relevant threshold probabilities against the default “treat-all” and “treat-none” strategies (14).

### Webpage deployment tool based on Streamlit framework

To enhance the clinical utility of this model, the optimal prediction model was deployed as a web application using the Streamlit Python-based framework. The application interface allows users, such as clinicians or patients, to conveniently input key clinical features, the web application automatically calculates the probability of Aortic Atherosclerosis. To ensure transparency, each prediction is accompanied by model-agnostic explainable outputs: SHAP force plots (shap package) demonstrating feature contributions to the prediction, and LIME explanations (lime package) visualizing local decision rules with feature weightings.

### Statistical analysis

Data analysis was conducted in DecisionLinnc 1.0, an integrated platform that supports multiple programming languages for data processing, analytical workflows, and machine learning model development via a visual interface (15). Continuous variables were tested for normality using the Shapiro–Wilk test. For comparisons between two groups, normally distributed data are presented as mean ± standard deviation (SD) and compared using independent *t*-tests; non-normally distributed data are presented as median (interquartile range, IQR) and compared using Mann–Whitney *U* tests. For comparisons among three or more groups, normally distributed data were compared using one-way analysis of variance (ANOVA), and if the overall ANOVA result was significant, *post-hoc* pairwise comparisons were performed using Tukey's honestly significant difference (HSD) test. For non-normally distributed data involving three or more groups, the Kruskal–Wallis *H* test was used, followed by Dunn's test with Bonferroni correction for significant *post-hoc* comparisons. Categorical variables are presented as number (*n*) and percentage (%), and comparisons were performed using the chi-square test or Fisher's exact test when the expected frequency in any cell was less than 5. A *P* value of less than 0.05 was considered statistically significant.

## Results

### Characteristics of the study participants

This study ultimately enrolled 362 individuals undergoing physical examination. The model development cohort consisted of 261 participants from the Health Management Centre of the First Affiliated Hospital of Anhui Medical University, who were randomly allocated to a training set (*n* = 182) and a testing set (*n* = 79) in a 7:3 ratio. Table 1 summarises the baseline characteristics of all participants. Inter-group comparisons revealed that the aortic sclerosis group exhibited significantly higher levels of Age, BMI, urea, FBG, SBP, DBP, and MAP, as well as elevated prevalence rates of diabetes and hypertension. In contrast, GFR and PLT were significantly reduced (all *P* < 0.05). The baseline characteristics of the training, testing, and validation sets are summarized in Supplementary Table S1.

**Table 1:** Baseline characteristics of the study participants according to the aortic arteriosclerosis.

Variable	Overall (*n* = 362)	No aortic arteriosclerosis group (*n* = 244)	Aortic arteriosclerosis group (*n* = 118)	*P*-value
Age (years)	52 (30–63)	44 (27–55)	63 (56–72)	<0.001
Gender (F/M)	158/204	113/131	45/73	0.142
BMI (kg/m^2^)	23.28 (21.23–25.26)	23.01 (20.81–24.92)	24.12 (21.87–26.30)	0.008
ALT (IU/L)	19 (14–28)	18 (12–29)	21 (15–27)	0.537
AST (IU/L)	22 (18–26)	21 (17–25)	23 (20–26)	0.139
GFR [mL·min^−1^·(1.73 m^2^)^−1^]	104 (94–118)	112 (100–123)	95 (86–102)	<0.001
Creatinine (μmol/L)	70.10 (59.60–80.40)	70.10 (59.10–80.10)	69.95 (61.25–80.45)	0.326
Urea (mmol/L)	5.16 (4.20–5.96)	4.92 (4.03–5.70)	5.58 (4.57–6.61)	<0.001
UA (μmol/L)	353 (293–415)	355 (291–418)	344 (297–410)	0.657
RBC (×10^12^/L)	4.69 ± 0.50	4.71 ± 0.52	4.64 ± 0.47	0.174
HGB (g/L)	143 (130–151)	144 (128–151)	142 (132–151)	0.458
PLT (×10^9^/L)	215 (177–255)	222 (187–259)	200 (170–239)	0.009
FBG (mmol/L)	5.14 (4.85–5.92)	5.01 (4.78–5.32)	6.15 (5.17–6.98)	<0.001
LDL (mmol/L)	3.10 (2.58–3.58)	3.02 (2.58–3.50)	3.28 (2.56–3.70)	0.135
TG (mmol/L)	1.22 (0.89–1.79)	1.17 (0.88–1.71)	1.35 (0.94–1.88)	0.096
HDL (mmol/L)	1.38 (1.22–1.58)	1.36 (1.21–1.57)	1.43 (1.26–1.59)	0.347
TC (mmol/L)	4.82 (4.17–5.44)	4.65 (4.14–5.37)	4.96 (4.19–5.59)	0.070
SBP (mmHg)	124 (114–133)	118 (110–127)	135 (129–145)	<0.001
DBP (mmHg)	75 (69–82)	73 (68–78)	82 (75–90)	<0.001
MAP (mmHg)	92.43 ± 10.85	88.44 ± 9.27	100.68 ± 9.12	<0.001
cfPWV (m/s)	9.24 (8.25–10.52)	8.75 (7.89–9.29)	11.12 (10.66–12.10)	<0.001
Hypertension (%)	73 (20.17%)	13 (5.33%)	60 (50.85%)	<0.001
DM (%)	27 (7.58%)	5 (2.08%)	22 (18.97%)	<0.001
Smoke (%)	59 (16.30%)	40 (16.39%)	19 (16.10%)	0.944

BMI, body mass index; ALT, alanine aminotransferase; AST, aspartate aminotransferase; GFR, glomerular filtration rate; UA, uric acid; RBC, red blood cell count; HGB, hemoglobin; PLT, platelet count; FBG, fasting blood glucose; LDL, low-density lipoprotein; TG, triglycerides; HDL, high-density lipoprotein; TC, total cholesterol; SBP, systolic blood pressure; DBP, diastolic blood pressure; MAP, mean arterial pressure; DM, diabetes mellitus.

### Missing data

Across all variables, missingness ranged from 0% to 22% (Supplementary Figure S1). Two variables (Eosinophil and Basophil) exceeded the prespecified 20% missingness threshold and were therefore removed. For all variables with missingness ≤20%, missing values were addressed using the imputation strategies described in the Methods, ensuring that no predictor variables remained incomplete. The outcome variable was not imputed. For outlier management, plausible extreme values identified via the interquartile range (IQR) method were retained, whereas implausible values were set as missing and subsequently imputed.

### Feature selection

The features selected by each feature selection method are summarized in Table 2. The univariate logistic regression method (Supplementary Table S2) identified five significant variables: MAP, Age, BMI, FBG, and GFR. The RFE method (Supplementary Figure S2C) selected seven features: MAP, Age, FBG, GFR, AST, PLT, and HDL-C. The LASSO regression (Supplementary Figure S2B), optimized by the lambda.min criterion, retained eleven predictors: MAP, Age, BMI, FBG, GFR, AST, Urea, UA, HGB, HDL-C, and TC. The final feature set, defined as the union of variables selected by at least two methods, comprised seven features: MAP, Age, BMI, FBG, GFR, AST, and HDL-C, which were used for subsequent model development.

**Table 2:** Feature union from four feature selection methods.

Methods	Features
Univariable regression	MAP, Age, BMI, FBG, GFR
RFE	MAP, Age, FBG, GFR, AST, PLT, HDL-C
LASSO	MAP, Age, BMI, FBG, GFR, AST, Urea, UA, HGB, HDL-C, TC
Union (candidate set)	MAP, Age, BMI, FBG, GFR, AST, HDL-C

RFE, recursive feature elimination; LASSO, least absolute shrinkage and selection operator.

To further assess potential multicollinearity among the selected predictors, Variance Inflation Factor (VIF) analysis was performed. As shown in Table 3, all VIF values were below 2, indicating the absence of problematic multicollinearity and confirming that all selected variables were suitable for inclusion in the subsequent modeling procedures.

**Table 3:** Variance inflation factor (VIF).

Variable	VIF
MAP	1.605
Age	1.509
BMI	1.148
FBG	1.343
GFR	1.399
AST	1.370
HDL-C	1.537

### Hyperparameter optimization

Using the seven selected predictors, we constructed six machine learning models. Hyperparameter optimisation was conducted on the training set via grid search coupled with 10-fold cross-validation (random seed = 42) to maximise model performance while mitigating overfitting risk. For each hyperparameter configuration, the average performance across all 5 folds was computed, and the configuration yielding the best performance was selected.

### Performance of ML models in test set

**Figure 3:**
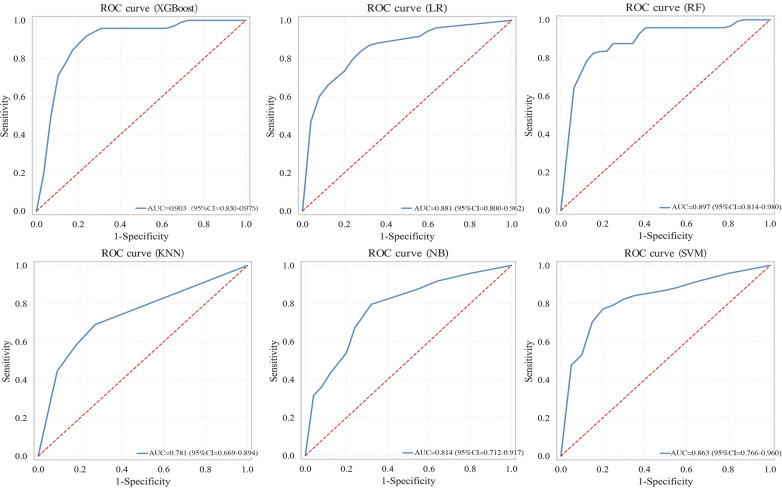
ROC curves comparing multiple machine learning models on test set.

**Figure 4:**
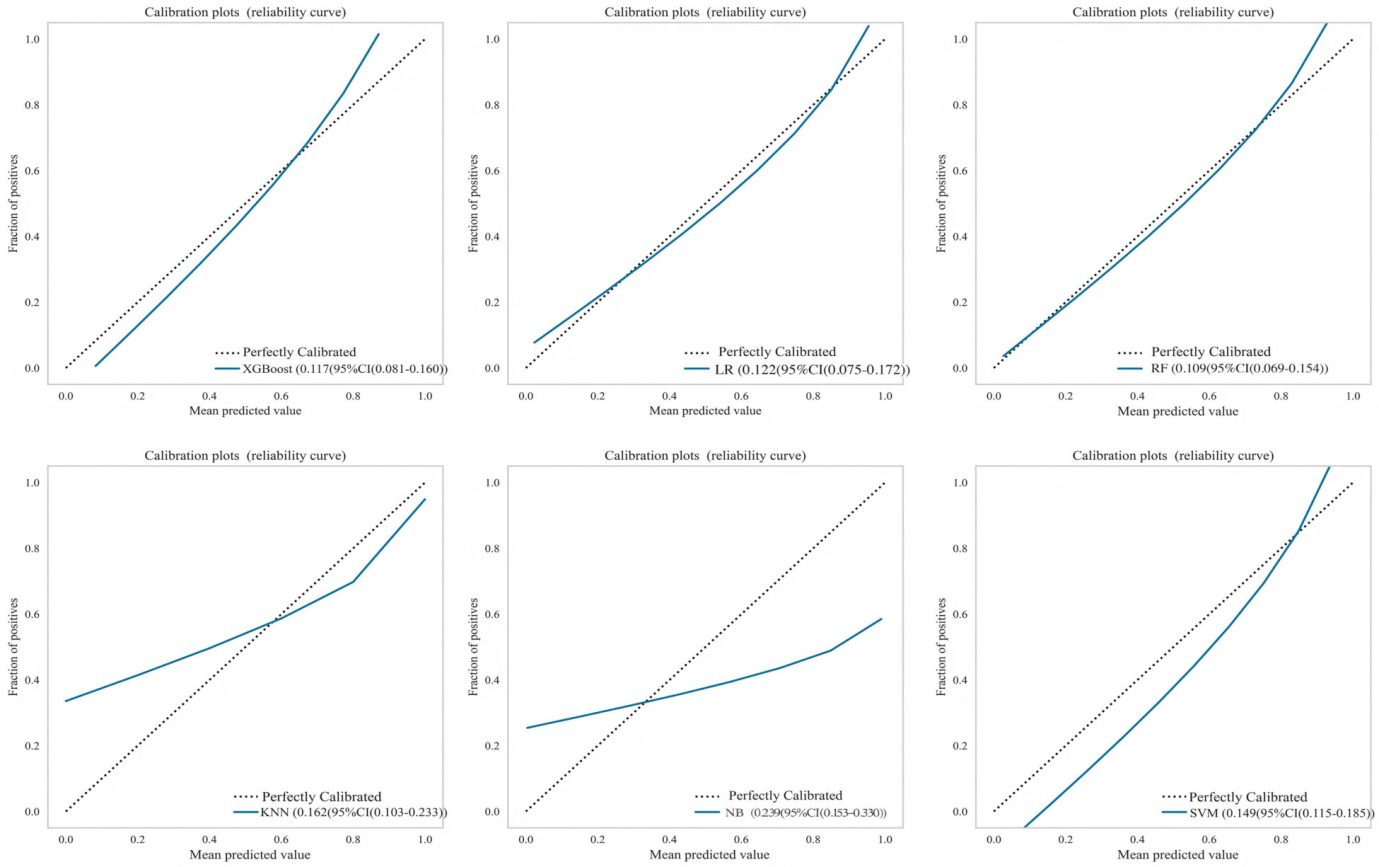
Calibration curves for multiple machine learning models on test set.

**Figure 5:**
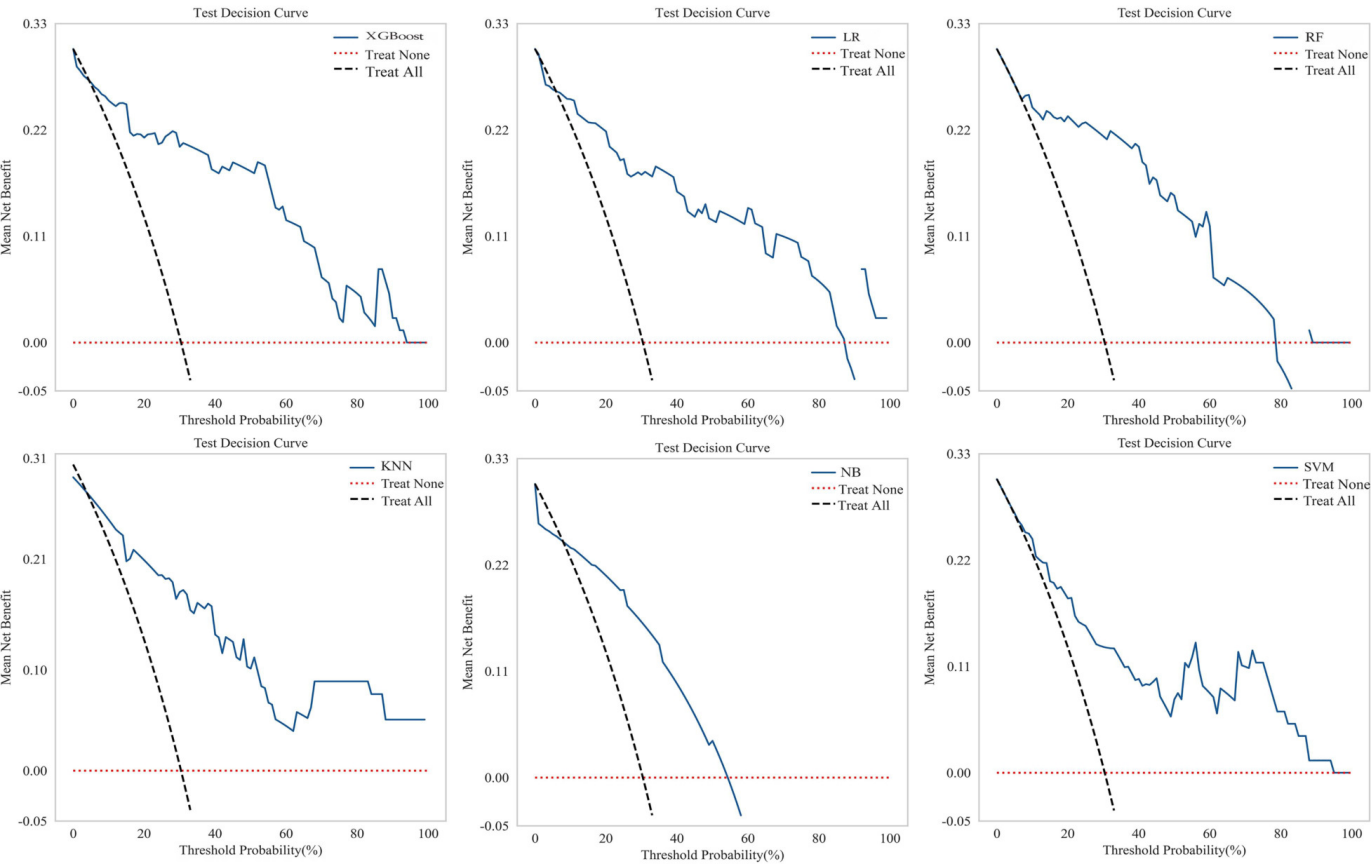
Decision curves for multiple machine learning models on test set.

Six machine learning models were evaluated on the test set (Table 4). The XGBoost model demonstrated superior overall performance, achieving an AUC of 0.903 (95% CI: 0.830–0.975), accuracy of 0.848, sensitivity of 0.750, specificity of 0.891, *F*1-score of 0.750, PPV of 0.750, and NPV of 0.891. ROC curves, calibration curves, and decision curve analyses for all models are presented in Figures 3–5, respectively.

**Table 4:** Performance of the machine learning models for predicting aortic arteriosclerosis in test set.

Model	AUC (95% CI)	Accuracy	Recall	Specificity	*F*1-Score	PPV	NPV
LR	0.881 (0.800–0.962)	0.835	0.583	0.945	0.683	0.824	0.839
RF	0.897 (0.814–0.980)	0.835	0.542	0.964	0.667	0.867	0.828
XGBoost	**0.903 (0.830–0.975)**	**0.848**	**0.750**	**0.891**	**0.750**	**0.750**	**0.891**
NB	0.814 (0.712–0.917)	0.709	0.875	0.636	0.646	0.512	0.921
SVM	0.863 (0.766–0.960)	0.747	0.792	0.727	0.655	0.559	0.889
KNN	0.781 (0.669–0.894)	0.785	0.333	0.982	0.485	0.889	0.771

Note: Bold indicates the model (XGBoost) that was ultimately selected.

### Internal validation

**Figure 6:**
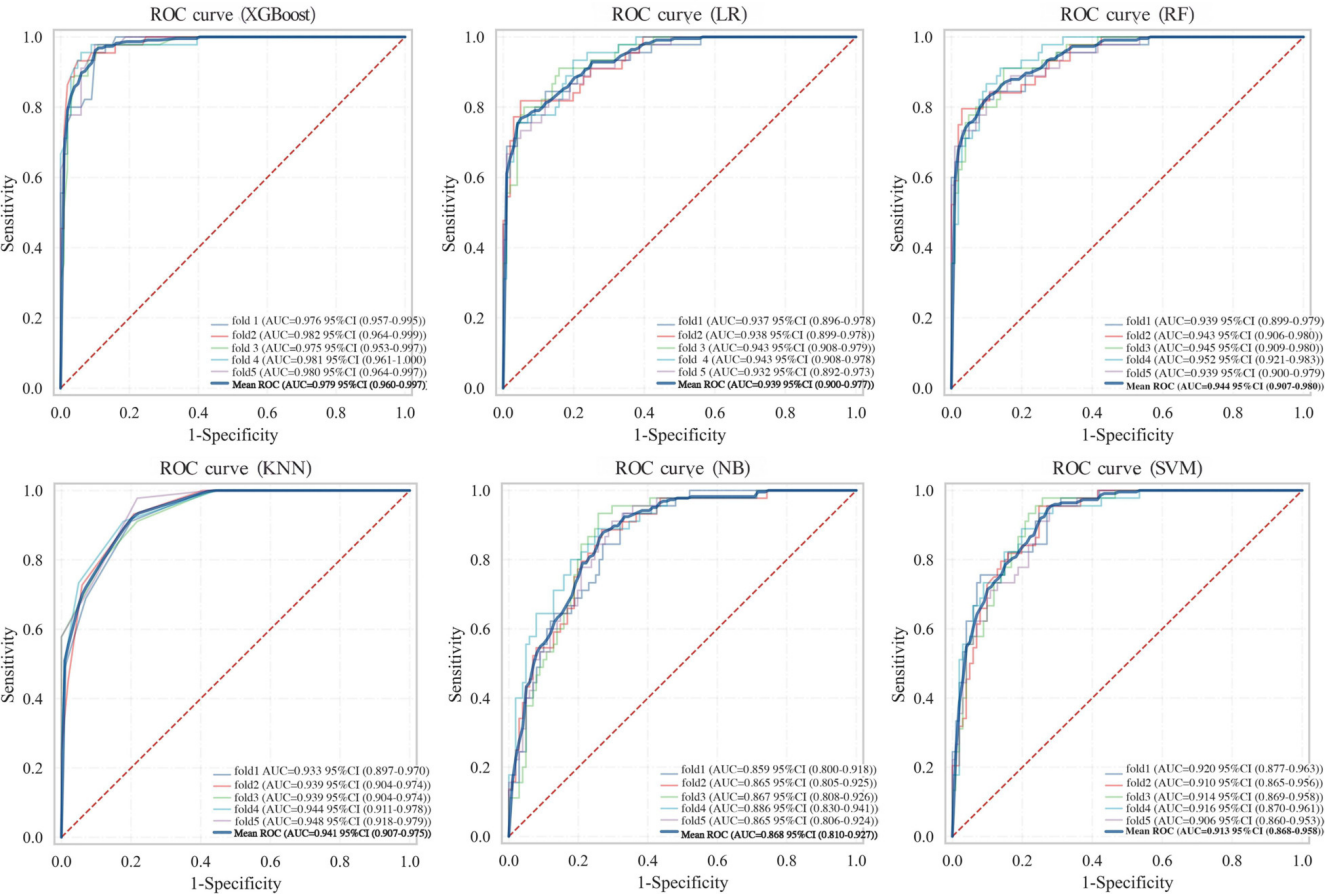
ROC curves of six machine learning models from 5-fold cross-validation on the training set.

To assess the robustness and generalisation capability of the XGBoost model, 5-fold cross-validation was performed on the training set using the optimised hyperparameters. In each iteration, the model was trained on 4/5 of the data and validated on the remaining 1/5. Despite minor variations across different data partitions, the XGBoost model achieved a mean AUC of 0.979 (95% CI: 0.960–0.997), demonstrating robust performance across all data partitions (Table 5). The overlaid ROC curves for all 5 folds (Figure 6) further illustrate the stability of the model's discriminative ability across different training-validation splits.

**Table 5:** Performance of the XGBoost model evaluated by 5-fold cross-validation on the training set.

Model	AUC	Accuracy	Recall	Specificity	*F*1-Score	PPV	NPV
XGBoost_1Test	0.938	0.806	0.500	0.958	0.632	0.857	0.851
XGBoost_2Test	0.955	0.889	0.778	0.926	0.778	0.778	0.937
XGBoost_3Test	0.946	0.861	0.846	0.870	0.815	0.786	0.905
XGBoost_4Test	0.946	0.917	0.846	0.957	0.880	0.917	0.941
XGBoost_5Test	0.865	0.861	0.800	0.885	0.762	0.727	0.923
Mean_scores	0.930	0.867	0.754	0.919	0.773	0.813	0.911

### External validation

**Figure 7:**
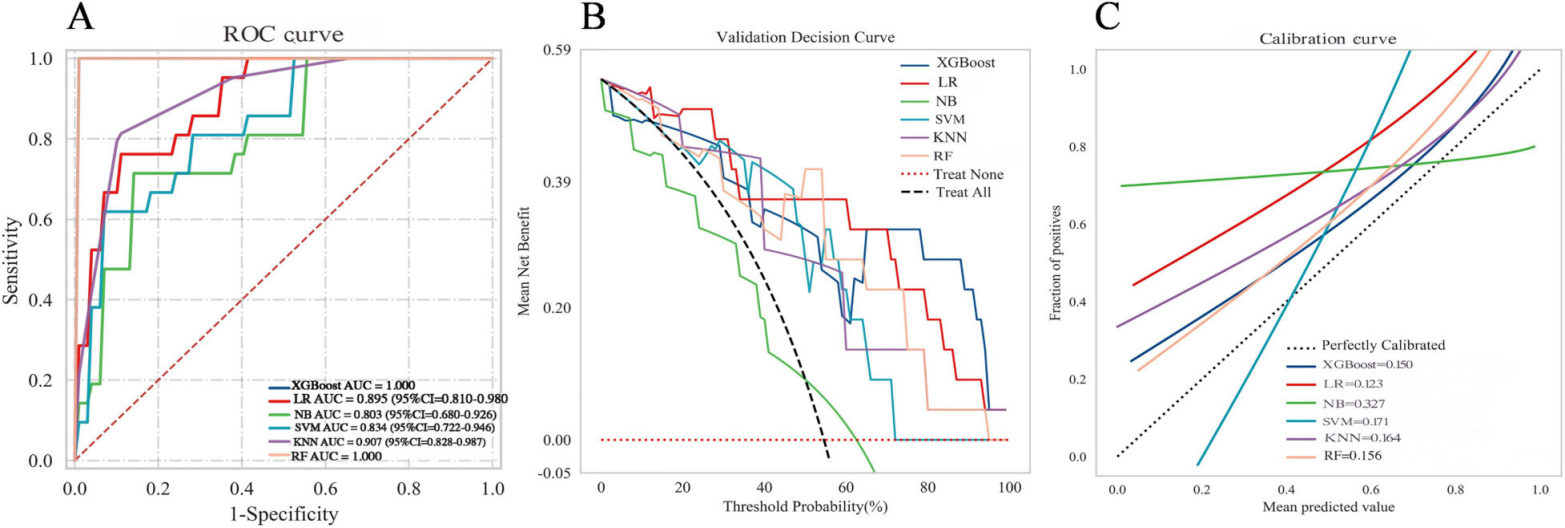
Comprehensive performance evaluation of the predictive model on the external validation set **(A)** receiver operating characteristic (ROC) curves; **(B)** decision curve analysis (DCA); **(C)** calibration curve.

An external validation was performed using an independent cohort from a different institution to assess the model's generalizability. As shown in Figure 7A, the XGBoost model demonstrated superior discriminative ability, achieving a perfect AUC of 1.000. The decision curve analysis (Figure 7B) confirmed that XGBoost provided a higher net clinical benefit across most threshold probabilities compared to “treat all” or “treat none” strategies, supporting its clinical utility for risk-stratified decision-making in aortic sclerosis management. Calibration curves (Figure 7C) revealed that XGBoost and LR exhibited better agreement between predicted and observed risks than other models. To provide quantitative calibration metrics, model-agnostic calibration errors were calculated. LR showed the lowest error (0.123), followed by XGBoost (0.150). Considering its exceptional discrimination and satisfactory calibration, the XGBoost model was selected as the final model for deployment.

### Model interpretability

**Figure 8:**
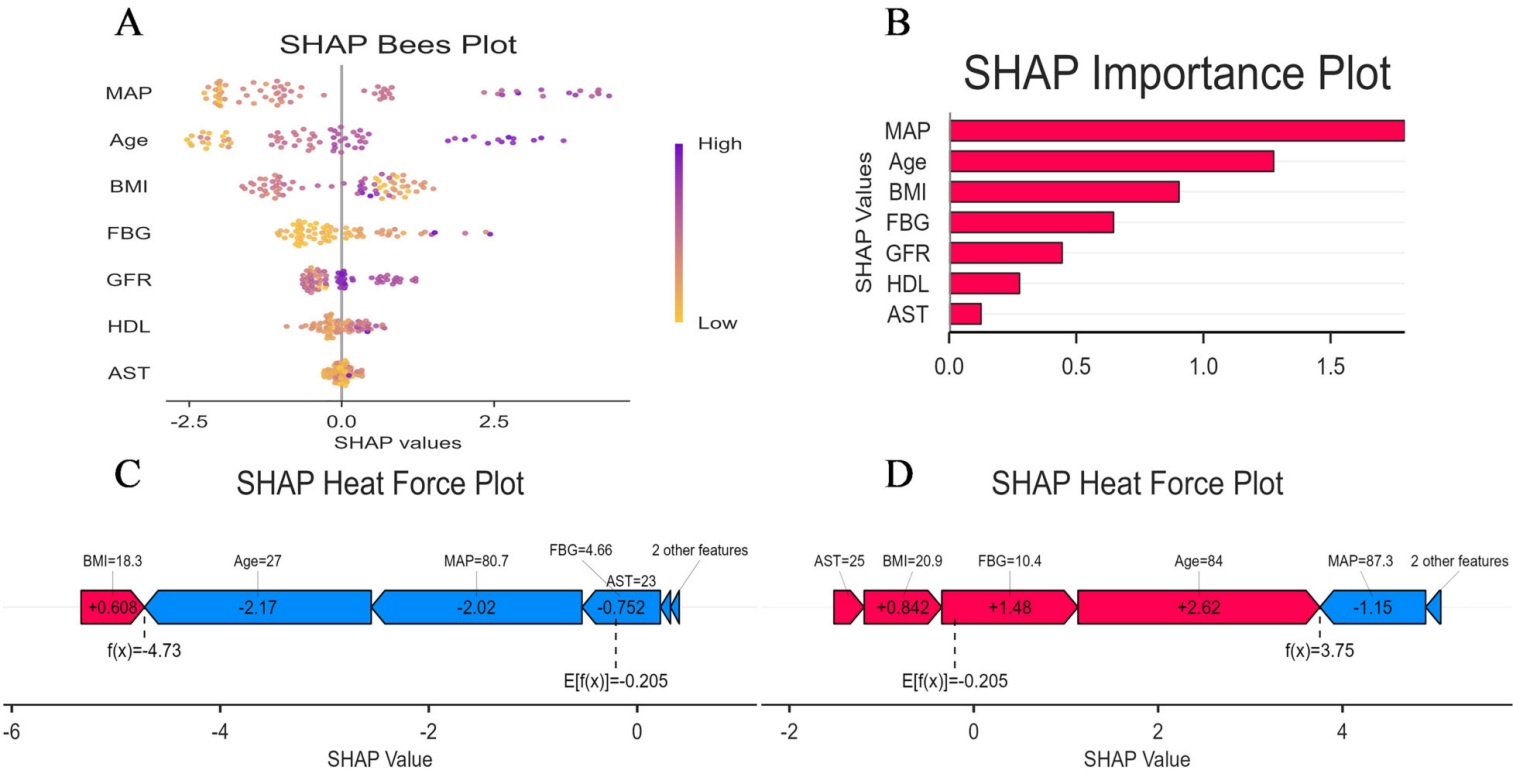
Model interpretation using SHAP: **(A)** global interpretation: beeswarm plot of SHAP value distributions for each feature. Point color represents the feature value (orange: low, purple: high); **(B)** global interpretation: feature importance ranking based on mean absolute SHAP values; **(C)** local interpretation: waterfall plot explaining the prediction for a representative low-risk individual; **(D)** local interpretation: waterfall plot explaining the prediction for a representative high-risk individual.

To enhance the XGBoost model transparency and facilitate clinical adoption, we conducted a comprehensive interpretability analysis using SHAP on the external validation cohort. The SHAP summary bar plot (Figure 8A) quantified global feature importance by mean absolute SHAP values, identifying MAP as the most influential predictor of aortic sclerosis, followed in descending order by FBG, Age, BMI, GFR, AST and HDL-C. The SHAP beeswarm plot (Figure 8B) further elucidated the directional relationship between feature values and model predictions. Higher values of MAP, FBG, Age BMI (depicted in purple) were consistently associated with positive SHAP values, indicating risk-increasing contributions. Conversely, elevated GFR and AST levels corresponded to negative SHAP values, suggesting a protective association with aortic sclerosis risk.

SHAP force plots provided granular, case-specific interpretations of model predictions (Figures 8C,D). In these visualisations, the base value *E*[*f*(*x*)] represents the mean predicted log-odds across the validation cohort, while the output value *f*(*x*) denotes the individual prediction. Feature contributions are colour-coded: red arrows indicate risk-increasing effects (positive SHAP values), and blue arrows indicate risk-decreasing effects (negative SHAP values), with arrow length proportional to contribution magnitude. Figure 8C illustrates a representative low-risk case with *f*(*x*) = −5.6. Here, FBG (5.03 mmol/L) and low MAP (75.3 mmHg) contributed substantial negative SHAP values of −1.67 and −1.58, respectively, driving the prediction well below the population baseline risk. In contrast, Figure 8D demonstrates a high-risk case with *f*(*x*) = 3.3, where elevated FBG (9 mmol/L) and BMI (26.3 kg/m^2^) contributed positive SHAP values of +2.59 and +0.944, respectively, substantially elevating the predicted probability of aortic sclerosis.

### Convenient application for clinical utility

**Figure 9:**
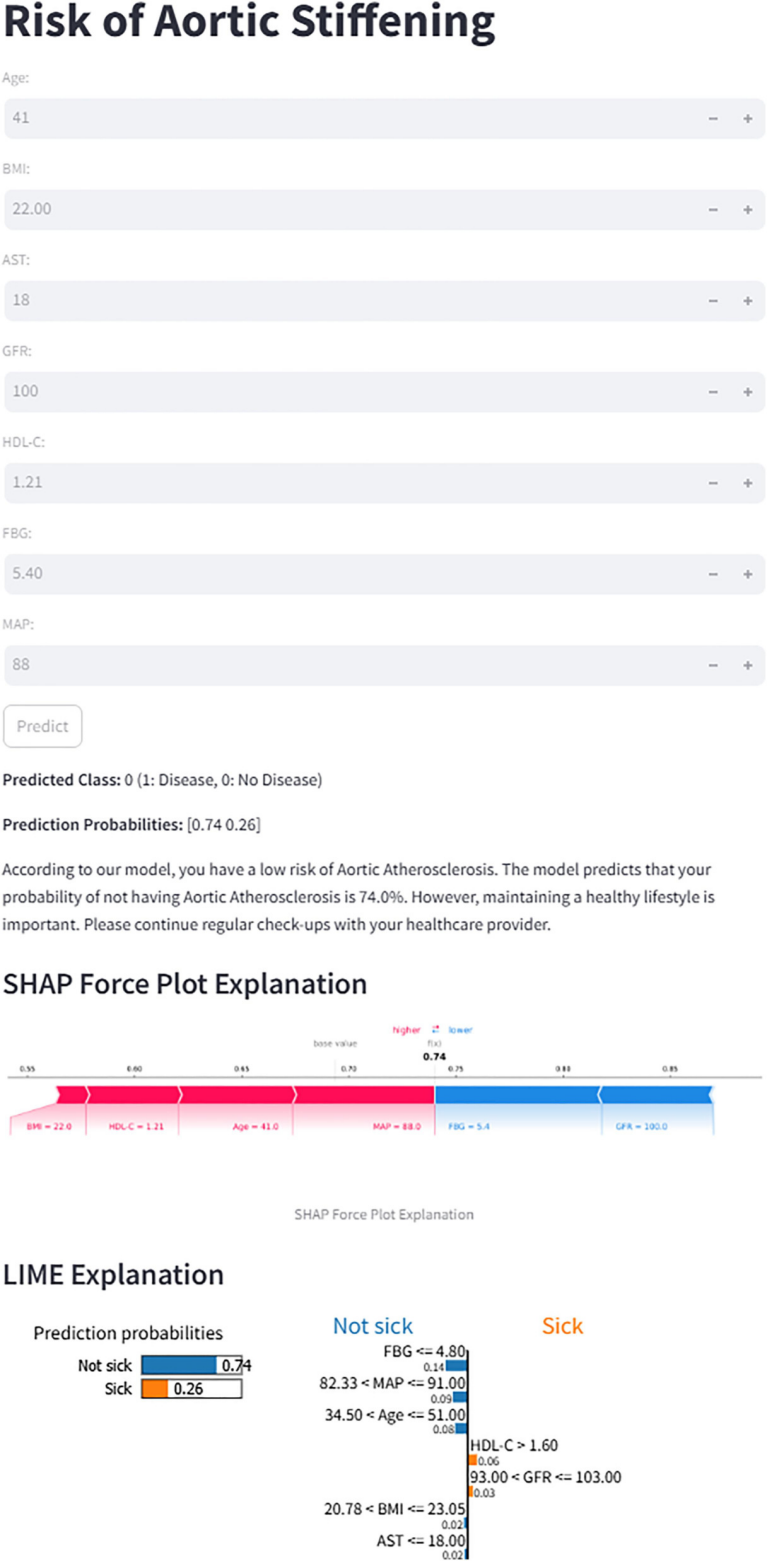
Web interface for the XGBoost prediction model. The application calculates the risk of aortic stiffening based on seven input features. For the displayed case, the model predicts a 74% probability of “No Disease”. Both SHAP and LIME explanations are provided to illustrate the contribution of individual features to the prediction.

The final prediction model was deployed as a publicly accessible web application to facilitate clinical utility (Figure 9). The application requires users to input the actual values of the seven clinical features used in the model (Age, BMI, AST, GFR, HDL-C, FBG, and MAP). Upon submission, it automatically generates a personalized prediction of aortic stiffeningrisk for an individual patient. To enhance transparency and interpretability, the interface integrates two advanced visualization techniques. First, an accompanying SHAP force plot is displayed, illustrating the contribution of each feature to the specific prediction score; it visualizes how factors such as BMI and HDL-C drive the model output from the base value. Second, a LIME plot is provided to further decode the decision-making process. The LIME explanation categorizes feature contributions into “Not Sick” and “Sick”, displaying specific probability weights and feature thresholds (e.g., FBG ranges or Age intervals). As shown in the interface, the model's prediction for the sample user yielded a probability of 0.74 for being “Not sick”. This result was directly communicated to the users as “According to our model, you have a low risk of Aortic Atherosclerosis.” The application is freely available at: https://lgezijo5wyivqrnt2zrkcm.streamlit.app/.

## Discussion

Aortic stiffness is an independent predictor of cardiovascular events; however, the gold standard measurement, cfPWV, faces equipment and technical limitations in large-scale screening within health management institutions. This study innovatively developed a machine learning model using routine health examination parameters to screen for aortic stiffness risk. A multi-strategy ensemble approach was employed, integrating Three feature selection strategies—univariate logistic regression, LASSO, and RFE—to identify seven core predictive features from candidate variables. Through systematic comparison of six machine learning algorithms, the XGBoost model demonstrated optimal discriminative performance. The model demonstrated robust stability and generalizability in both internal (five-fold cross-validation) and external validation. Calibration curves confirmed strong concordance between predicted probabilities and actual risk, while decision curve analysis demonstrated significant clinical net benefit. SHAP value analysis provided a clear interpretability framework. This model, relying solely on readily accessible clinical parameters, offers a cost-effective and practical alternative for health management institutions lacking cfPWV measurement capabilities, demonstrating promising clinical application prospects.

Among the seven core predictors identified in this study, MAP demonstrated the strongest predictive power, a finding highly consistent with the pathophysiological mechanisms of aortic stiffening. Aortic stiffening leads to impaired vascular elastic function, characterised by elevated systolic pressure, reduced diastolic pressure, and widened pulse pressure (16). These hemodynamic alterations increase cardiac afterload and compromise coronary and cerebrovascular perfusion, thereby elevating the risk of myocardial infarction and stroke (3). MAP reflects the integrated effects of cardiac output and peripheral resistance, representing the sustained mechanical stress imposed on the vascular wall (17). Evidence suggests that persistently elevated MAP subjects the vascular wall to excessive mechanical tension, activating vascular smooth muscle cell proliferation and extracellular matrix remodelling.

Furthermore, shear stress alterations induced by elevated MAP impair endothelial function, initiating inflammatory responses and atherosclerotic processes (18, 19). Previous studies have confirmed that MAP is superior to conventional blood pressure indices in predicting aortic stiffening. Compared with systolic or diastolic pressure alone, MAP more accurately reflects the sustained pressure load on the vascular wall, demonstrating higher sensitivity and specificity in early identification of aortic stiffening risk (20). As the gold standard for assessing aortic stiffness, cfPWV increases significantly with Age, typically ranging from 6 to 8 m/s in individuals aged 20–30 years and rising to 12–15 m/s in those over 70 years (21). Age serves not only as an independent risk factor for arterial stiffening but, more importantly, modulates the strength of association between MAP and aortic stiffness (22). Specifically, in young individuals with robust vascular compensatory capacity, the effect of MAP on arterial stiffening is relatively modest. During middle Age, when arterial stiffening progresses rapidly, the predictive value of MAP becomes most prominent, suggesting a critical window for preventive intervention. Conversely, in elderly individuals where arterial stiffening is already prevalent, the incremental predictive value of MAP may be attenuated (23). These findings underscore the necessity of age-stratified prevention of aortic stiffness, identifying middle Age as the critical window for intensive MAP control. Clinical practice should develop personalised MAP management strategies tailored to age-specific vascular phenotypes and individual risk characteristics.

In addition to MAP and Age, this study identified several biomarkers significantly associated with aortic stiffness. The positive correlation between BMI and arterial stiffness is mediated primarily through chronic low-grade inflammation, insulin resistance, and oxidative stress. In the obese state, pro-inflammatory cytokines (such as IL-6 and TNF-α) secreted by adipose tissue and imbalanced adipokines collectively promote vascular remodelling. A meta-analysis by Jamialahmadi et al. (24) demonstrated a significant reduction in cfPWV of 1.12 m/s (95% CI: 0.67–1.57) following bariatric surgery, providing interventional evidence for the causal relationship between obesity and arterial stiffening. Hyperglycemia promotes arterial stiffening through multiple interconnected pathophysiological mechanisms. Sustained hyperglycemic conditions induce non-enzymatic glycation to form advanced glycation end products (AGEs), which activate inflammatory signaling pathways upon binding to their receptor RAGE; concurrently, activation of protein kinase C (PKC) signaling cascades upregulates pro-inflammatory cytokines and adhesion molecules, exacerbating vascular wall inflammation; additionally, hyperglycemia increases reactive oxygen species (ROS) generation, leading to oxidative stress imbalance and endothelial dysfunction; collectively, these pathological alterations promote vascular smooth muscle cell phenotypic switching from contractile to synthetic phenotype, accelerating vascular remodeling and arterial stiffening progression (25–27). In a prospective study of healthy men, Sang et al. (28). It was found that even at the prediabetic stage, a mild elevation of fasting glucose (5.6–6.9 mmol/L) was significantly associated with arterial stiffness progression. This suggests that glucose-mediated vascular damage may precede the clinical diagnosis of diabetes and underscores the importance of early glycemic management.

In our study, SHAP analysis did not reveal the traditionally expected inverse relationship between HDL-C levels and aortic stiffness risk. This finding aligns with the emerging “HDL paradox” in cardiovascular research (29). While HDL-C has been conventionally considered cardioprotective through reverse cholesterol transport and anti-inflammatory mechanisms (30), accumulating evidence suggests that HDL-C quality and functionality are more important than quantity (31, 32). In pathological conditions such as diabetes and metabolic syndrome, HDL-C particles can undergo functional impairment and transform into pro-inflammatory HDL-C, losing their protective properties or even exerting pro-atherogenic effects (33). Casula et al. demonstrated that extremely high HDL-C levels may paradoxically associate with increased cardiovascular risk due to HDL-C particle heterogeneity and functional abnormalities (34). Moreover, Our study revealed a negative association between GFR and aortic stiffness. This finding reflects the well-established bidirectional relationship between renal dysfunction and arterial stiffness (35). Reduced GFR promotes arterial stiffness through: (1) mineral metabolism disorders with calcium-phosphate imbalance and elevated FGF-23 accelerating vascular calcification (36); (2) chronic inflammation with uremic toxin accumulation exacerbating endothelial damage; (3) oxidative stress promoting vascular remodeling (37); and (4) sodium-water retention intensifying arterial wall stress. Conversely, increased aortic stiffness elevates pulse pressure, transmitting excessive pulsatile stress to renal microcirculation and causing glomerular injury, ultimately leading to GFR decline (38). Notably, even mild GFR reduction in non-CKD populations correlates with arterial stiffness (39). These findings emphasize the importance of early renal function monitoring in arterial stiffness prevention and management.

This study also found a negative correlation between AST and aortic stiffness, which is inconsistent with some previous studies reporting positive or null associations. This discrepancy may stem from several factors. First, the relationship between AST and arterial stiffness may be nonlinear, exhibiting different biological effects across concentration ranges—pathological elevation (reflecting hepatic injury) may correlate positively with arterial stiffness. In contrast, relatively higher levels within the normal range might reflect tissue metabolic activity. Second, differences in study population characteristics may influence results; in our relatively healthy cohort, AST elevation likely reflects physiological variation rather than pathological states. Therefore, the value of AST in arterial stiffness risk assessment requires further investigation, and clinical interpretation should integrate other liver function indices and the individual clinical context.

Traditional statistical methods for constructing predictive models offer good interpretability but may have limitations in capturing complex nonlinear relationships. In contrast, ensemble learning methods (such as random forest and gradient boosting) can capture complex variable interactions but face challenges, including overfitting risk and reduced interpretability. Therefore, a systematic empirical comparison is necessary to determine the optimal modelling strategy. In our study, we developed and compared six machine learning models, and the results demonstrated that the XGBoost model outperformed the other models in predictive performance. XGBoost's superior performance can be attributed to its gradient boosting framework with regularization techniques that effectively prevent overfitting and handle complex non-linear relationships in clinical data (40). Additionally, XGBoost provides interpretable feature importance rankings and demonstrates robust performance with imbalanced datasets, making it particularly suitable for cardiovascular risk prediction (41). To translate our predictive model into a practical clinical tool, we have developed an interactive web-based calculator utilizing the optimal XGBoost algorithm. This tool allows users (clinicians or individuals) to input routine hematological parameters and obtain an instant, individualized risk assessment for aortic stiffness. A representative output of the tool is demonstrated in the attached figure. For a hypothetical 41-year-old individual with normal biochemical profiles, the model predicted a low probability (26%) of aortic stiffness, classifying the case as “No Disease.”

Importantly, to enhance clinical trust and understanding, the web interface integrates two advanced model interpretation frameworks: SHAP and LIME explanations. The SHAP force plot visually quantifies and displays the contribution of each input feature (e.g., MAP, Age, HDL-C) towards pushing the final prediction towards either “Higher risk” or “Lower risk” for that specific individual. In the provided example, features like lower FBG and younger age contributed positively to the “low-risk” prediction. Simultaneously, the LIME explanation locally approximates the complex XGBoost model with an interpretable linear model, listing the most influential local features and their directional impact. For instance, it explicitly shows that “FBG ≤4.80” and “34.50 < Age ≤51.00” were the top rules supporting the “Not sick” classification. While the model provides a quantitative risk estimate, clinical decision-making should integrate this with comprehensive patient assessment. Therefore, even for cases predicted as low risk, the tool includes a cautionary note emphasizing the importance of maintaining a healthy lifestyle and adhering to regular health check-ups, as cardiovascular risk is dynamic and multifactorial.

## Conclusion

We present a web-based model designed to predict the risk of aortic stiffness from routine clinical biomarkers. It showed stable performance upon internal cross-validation and generalized effectively to an external cohort. This tool offers a viable, low-cost solution for initial screening, enabling aortic stiffness risk assessment in contexts without direct access to cfPWV. However, this study has several limitations. First, the sample size was relatively limited (training set *n* = 182, external validation set *n* = 101). Although the sample size satisfied the minimum requirement of events per variable (EPV >10) for logistic regression modeling (42), the relatively modest cohort may still constrain the precision of parameter estimates. This model provides a starting point; future large-scale, multi-center studies are the necessary next step to enhance its generalizability across diverse populations. Second, as a cross-sectional study, this research only evaluated the model's ability to predict current aortic stiffness status; its predictive efficacy for the dynamic progression of aortic stiffness and long-term cardiovascular events needs to be validated through prospective cohort studies. Third, the participants were primarily recruited from a health examination population in a single region, which may introduce selection bias and limit the generalizability of the findings.

Future research should focus on expanding the sample size through multi-centre collaborations, particularly by incorporating data from different regions and ethnic populations to enhance the model's generalizability. Additionally, prospective cohort studies are warranted to evaluate the model's predictive value for aortic stiffness progression and cardiovascular events.

## Data Availability

The original contributions presented in the study are included in the article/Supplementary Material, further inquiries can be directed to the corresponding author.
