# Supplementary figures and images for: Development of an interpretable machine learning model for early prediction of aortic stiffness risk in health examination populations
Source: Front Cardiovasc Med. 2026 Jan 7;12:1730409. doi: 10.3389/fcvm.2025.1730409 (PMC12819839; doi:10.3389/fcvm.2025.1730409)

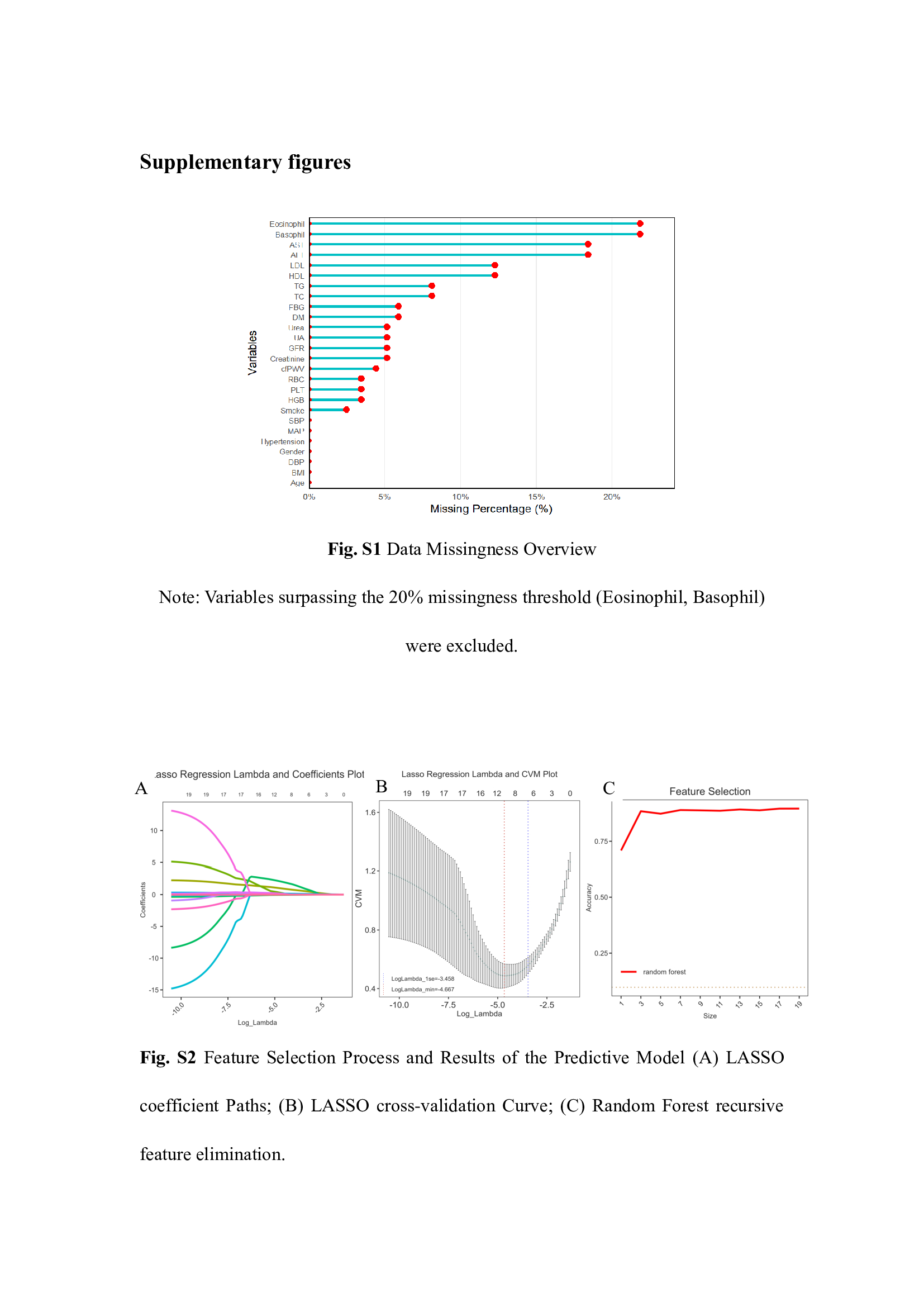

Supplement: Supplementary file 1 [file Supplementaryfile1.jpeg]
