# Supplementary material for: Development of an interpretable machine learning model for early prediction of aortic stiffness risk in health examination populations
Source: Front Cardiovasc Med. 2026 Jan 7;12:1730409. doi: 10.3389/fcvm.2025.1730409 (PMC12819839; doi:10.3389/fcvm.2025.1730409)
Supplement: Supplementary file 2 [file Supplementaryfile2.docx]

**Supplementary tables**

**Table S1** Baseline Data for the training, testing, and validation datasets

| **Variable** | **Overall**  **（n= 362）** | **Training set（n= 182）** | **Testing set（n= 79）** | **Validation set（n= 101）** | ***P*-value** |
| --- | --- | --- | --- | --- | --- |
| Age (years) | 53 (30-64) | 52 (31-64) | 50 (30- 59) | 56 (30 - 68) | 0.132 |
| Gender(F/M) | 158/204 | 87 /95 | 29/50 | 42/59 | 0.079 |
| BMI（kg/m2） | 23.38 (21.33-25.10) | 23.43 (21.28-25.18) | 22.97  (21.37-25.15) | 23.42 (21.37-24.92) | 0.912 |
| ALT（IU/L） | 19 (13- 27) | 18 (13-27) | 19 (14-26) | 20 (14-29) | 0.732 |
| AST（IU/L） | 22 (18-26) | 22 (18-26) | 22 (18-27) | 22 (19-26) | 0.782 |
| GFR[mL·min-1·（1.73 m2）-1] | 104 (94- 117) | 104 (95 - 117) | 107 (97-118) | 100 (91-109)^b^ | 0.008 |
| Creatinine (umol/L) | 70.45 (59.60-80.50) | 70.85 (59.55-81.25) | 65.50 (57.30-77.30) | 72.50 (62.20-81.35) | 0.087 |
| Urea(mmol/L) | 5.19 (4.26-6.08) | 5.20  (4.30-6.06) | 4.79 (4.03-5.67)^a^ | 5.56  (4.62-6.61)^b^ | 0.005 |
| UA (umol/L) | 346  (290 - 413) | 353  (298- 419) | 331  (282 - 406) | 355  (297- 400) | 0.562 |
| RBC(×1012/L) | 4.67 ± 0.50 | 4.68 ± 0.51 | 4.66 ± 0.49 | 4.66 ± 0.50 | 0.956 |
| HGB（g/L） | 143  (130 - 151) | 142  (129 - 151) | 143  (130 - 152) | 143  (130 - 151) | 0.874 |
| PLT（×109/L） | 213 (176 - 254) | 214(182 - 255) | 219(179 - 265) | 202(166 - 236) | 0.102 |
| FBG (mmol/L) | 5.08  (4.78 - 5.50) | 5.03  (4.76 - 5.45) | 5.11  (4.79 - 5.40) | 5.10  (4.79 - 6.10) | 0.302 |
| LDL (mmol/L) | 3.03  (2.53 - 3.55) | 3.06  (2.61 - 3.58) | 3.00  (2.45 - 3.46) | 3.09  (2.50 - 3.49) | 0.538 |
| TG (mmol/L) | 1.21  (0.86 - 1.67) | 1.21  (0.87 - 1.67) | 1.16  (0.87 - 1.78) | 1.22  (0.82 - 1.64) | 0.847 |
| HDL (mmol/L) | 1.37  (1.22 - 1.58) | 1.40  (1.22 - 1.59) | 1.37  (1.24 - 1.59) | 1.34  (1.16 - 1.56) | 0.614 |
| TC (mmol/L) | 4.74  (4.11 - 5.39) | 4.83  (4.20 - 5.44) | 4.56  (3.96 - 5.26) | 4.76  (4.04 - 5.37) | 0.440 |
| SBP (mmHg) | 124  (114- 134) | 125  (114 - 134) | 123  (112 - 131) | 125  (117 - 137) | 0.277 |
| DBP (mmHg) | 75 (69 - 82) | 76 (69 - 83) | 75 (69 - 81) | 74 (70 - 82) | 0.896 |
| MAP (mmHg) | 92.36 ± 10.86 | 92.39 ± 11.25 | 91.76 ± 10.14 | 92.85 ± 10.73 | 0.821 |
| cfPWV (m/s) | 9.1 (8.0 - 10.5) | 9.1 (7.9 - 10.4) | 9.0 (8.0-10.2) | 8.9 (7.9 - 10.3) | 0.672 |
| Aortic Arteriosclerosis  (%) | 118 (32.60%) | 58 (31.87%) | 22 (27.85%) | 38 (37.62%) | 0.498 |
| DM(%) | 26 (7.18%) | 14 (7.69%) | 4 (5.06%) | 8 (8.42%) | 0.551 |
| Hypertension(%) | 87 (24.03%) | 44 (24.18%) | 19 (24.05%) | 24 (23.76%) | 0.859 |
| Smoke(%) | 65 (17.96%) | 33 (18.13%) | 13 (16.46%) | 19 (18.81%) | 0.697 |

Note: BMI, Body Mass Index; ALT, Alanine Aminotransferase; AST, Aspartate Aminotransferase; GFR, Glomerular Filtration Rate; UA, Uric Acid; RBC, Red Blood Cell count; HGB, Hemoglobin; PLT, Platelet count; FBG, Fasting Blood Glucose; LDL, Low-Density Lipoprotein; TG, Triglycerides; HDL, High-Density Lipoprotein; TC, Total Cholesterol; SBP, Systolic Blood Pressure; DBP, Diastolic Blood Pressure; MAP, Mean Arterial Pressure; DM, Diabetes Mellitus. ^a^ *P* < 0.05 vs. Training set; ^b^ *P* < 0.05 vs. Testing set.

**Table S2** Results of univariable logistic regression analysis for factors associated with aortic stiffness.​

| **Variable** | **β** | **SE** | **z_value** | ***P* value** | **OR** | **95%*CI*** |
| --- | --- | --- | --- | --- | --- | --- |
| Age | 0.113 | 0.018 | 6.134 | <0.001 | 1.120 | 1.084～1.166 |
| Gender | 0.381 | 0.321 | 1.184 | 0.236 | 1.463 | 0.782～2.768 |
| BMI | 0.044 | 0.047 | 0.943 | 0.345 | 1.045 | 0.953～1.147 |
| GFR | -0.094 | 0.016 | -5.851 | <0.001 | 0.911 | 0.88～0.937 |
| Creatinine | 0.010 | 0.011 | 0.880 | 0.379 | 1.010 | 0.988～1.033 |
| Urea | 0.247 | 0.117 | 2.115 | 0.034 | 1.280 | 1.020～1.617 |
| UA | -0.004 | 0.002 | -2.022 | 0.043 | 0.996 | 0.992～1.000 |
| RBC | -0.093 | 0.300 | -0.309 | 0.757 | 0.911 | 0.500～1.635 |
| HGB | 0.017 | 0.010 | 1.728 | 0.084 | 1.017 | 0.998～1.038 |
| PLT | -0.007 | 0.003 | -2.184 | 0.029 | 0.993 | 0.987～0.999 |
| LDL | 0.171 | 0.207 | 0.830 | 0.407 | 1.187 | 0.79～1.787 |
| TG | 0.097 | 0.164 | 0.594 | 0.553 | 1.102 | 0.789～1.521 |
| HDL | 1.469 | 0.581 | 2.530 | 0.011 | 4.346 | 1.419～14.033 |
| TC | 0.261 | 0.160 | 1.628 | 0.104 | 1.298 | 0.950～1.790 |
| Smoke | 0.080 | 0.425 | 0.188 | 0.851 | 1.083 | 0.455～2.446 |
| MAP | 0.154 | 0.025 | 6.238 | <0.001 | 1.166 | 1.115～1.229 |
| ALT | -0.002 | 0.008 | -0.237 | 0.813 | 0.998 | 0.981～1.013 |
| AST | 0.029 | 0.017 | 1.641 | 0.101 | 1.029 | 0.995～1.066 |
| FBG | 1.845 | 0.300 | 6.159 | <0.001 | 6.330 | 3.677～11.967 |
